# The development of the Helping your Anxious Child programme: a parent-mediated group intervention for parents of autistic children in South Asia

**DOI:** 10.1192/bji.2023.37

**Published:** 2024-05

**Authors:** Caitlin Kittridge, Priyanka Rob, Amy Fisher-Rogers, Tarana Anis, Udena Attygalle, Farzana Islam, Aditya Narain Sharma, Jacqui Rodgers

**Affiliations:** 1 Research Assistant, School of Psychology, Newcastle University, Newcastle Upon Tyne, UK; 2 Research Assistant, School of Psychology, Newcastle University, Newcastle Upon Tyne, UK; 3 Research Assistant, School of Psychology, Newcastle University, Newcastle Upon Tyne, UK; 4 Senior Child Psychologist, Child Development Centre, Evercare Hospital, Dhaka, Bangladesh; 5 Consultant Child and Adolescent Psychiatrist, Sirimavo Bandaranayake Children's Hospital, Peradeniya, Sri Lanka; 6 Specialist, Child Development Centre, Evercare Hospital, Dhaka, Bangladesh; 7 Clinical Senior Lecturer and Honorary Consultant in Child and Adolescent Psychiatry, Translational and Clinical Research Institute, Newcastle University, Newcastle upon Tyne, UK; 8 Professor of Psychology and Mental Health, Population Health Sciences Institute, Newcastle University, Newcastle upon Tyne, UK. Email jacqui.rodgers@newcastle.ac.uk

**Keywords:** Anxiety or fear-related disorders, autism spectrum disorders, low- and middle-income countries, neurodevelopmental disorders, psychosocial interventions

## Abstract

Autistic children are at increased risk of experiencing a range of mental health difficulties, including anxiety. A number of intervention programmes are now available in high-income countries to support autistic children. However, to date there are no evidence-based interventions to support families of such children in South Asia. Based on consultations with clinicians, researchers and parents in Bangladesh and Sri Lanka, we developed a culturally tailored two-session skills-based group programme for parents whose autistic children present with anxiety. This paper describes the process of creating this programme, to be delivered by mental health professionals.

Autism spectrum disorder (ASD) is a lifelong neurodevelopmental condition characterised by difficulties with social communication and social interaction, and restrictive and repetitive behaviours, interests or activities. Autistic children are at increased risk of experiencing a range of mental health difficulties, including anxiety, which has been found to affect around 40%.^1^ Anxiety can have a negative impact on a child's social development^2^ and academic attainment.^3^

A number of parent-led cognitive–behavioural therapy (CBT) programmes for typically developing children experiencing anxiety are available^4^ and in recent years programmes have been developed in high-income countries to address anxiety in autistic children (for a recent review see^5^). Anxiety in autism is particularly complex and may include autism-related features such as anxiety related to sensory differences, restrictive and repetitive behaviours, uncertainty and unusual phobias or idiosyncratic fears.^6^ Consequently, anxiety interventions for autistic children need to be tailored to meet their needs and may include modifications such as visual aids, significant behavioural components, emotional literacy work and incorporation of interests.^7^ Furthermore, parent-mediated approaches may be helpful in supporting generalisation of strategies across a range of contexts outside a clinical setting. Over the past 15 years or so a range of manualised interventions for autistic children experiencing anxiety have been developed in high-income countries.^8^ Anxiety in autism has also been identified as a research priority for the autism community globally,^9^ further highlighting the significant international importance. The high rates of anxiety in autistic children, limited availability of trained professionals in low- and middle-income countries (LMICs) and lack of an evidence-based intervention specifically to address anxiety experienced by children on the spectrum in LMICs all point towards a need for an intervention to be developed. The Helping your Anxious Child (HAC) programme was created to fill this treatment gap.

The HAC programme is underpinned by pre-existing frameworks from parent groups for anxious children developed in the West. HAC is designed to suit the needs of parents in South Asia, with adaptations designed in co-production with clinicians, researchers and parents of autistic children to ensure suitability and cultural appropriateness.^10^

## The Helping your Anxious Child programme

Helping your Anxious Child was developed through collaborations via the North East England South Asia Mental health Alliance (NEESAMA; www.NEESAMA.org). NEESAMA seeks to bring together international partners in the UK and South East Asia to improve research, training and clinical service delivery within mental health services. At an international summit in Dhaka in 2019 an unmet need for an intervention to support autistic children experiencing anxiety was identified. This resulted in the Helping your Anxious Child (HAC) programme being developed through partnerships from Sri Lanka, Bangladesh and the UK.

The HAC programme is a manualised parent-mediated group intervention to address anxiety experienced by autistic children, specifically in LMICs. Based on CBT techniques, HAC aims to increase understanding of anxiety symptoms and how they may relate to autism, to identify helpful and less helpful strategies in managing anxiety and, if developmentally appropriate, to promote the use of helpful strategies to increase children's ability to manage their anxiety and experience of stressful situations on their own. In addition to being cost-effective, a parent group programme was considered as it allows parents to teach children coping strategies across a range of settings and it has the potential to improve family and child outcomes.^11,12^

The HAC programme was designed following consultation with parents, researchers and clinicians in Sri Lanka and Bangladesh to discuss potential benefits and challenges of delivering a parent group programme in those countries. The collaboration also ensured that the programme was potentially feasible for delivery within existing services. Following consultation with clinicians in Bangladesh and Sri Lanka, examples of specific adaptions to the programme included the following: (a) delivering fewer, longer sessions than the number typically included in such programmes (this is because parents advised that it would be very challenging to attend for more than two sessions); (b) incorporating stress management techniques using methods and techniques that parents and clinicians advised that families may be familiar with, for example butterfly hugs and pranayama; (c) not including the term autism in the title of the programme, owing to concerns regarding stigma; (d) providing a range of culturally adapted case examples; (e) providing opportunity for parents to practise their skills on a variety of culturally appropriate case examples; (f) reducing the emphasis on homework, to reduce its burden; (g) running smaller parent groups such as groups of three; and (h) increasing the amount of interactive activities/experiential exercises.

The HAC programme comprises two 4-h parent group sessions with a recommended 5–10 parents per group, guided by two facilitators. The sessions provide a range of strategies for managing anxious and stressful situations and are individualised to meet the needs of each parent and their child. Materials for the delivery of the programme include a presentation for each of the sessions, a therapist manual and a parent handout. The programme components include: (a) psychoeducation on cognitive, behavioural, emotional and physical presentations of anxiety; (b) psychoeducation on parent-reported common anxiety triggers and on recognising anxiety triggers using the STAR framework (‘situation, trigger, anxiety, response’); (c) supporting children to develop emotional literacy; (d) working towards SMART (‘specific, measurable, achievable, relevant and time-bound’) goals; (e) drawing on a toolbox of strategies (physical/relaxation/sensory tools, social tools and thinking tools) to apply in example case studies, and reflecting on how parents can apply these in real-life situations; and (f) managing setbacks.

## Results of pilot HAC programmes and next steps

Pilot HAC programmes have now been delivered to 14 parents of autistic children in Sri Lanka and Bangladesh. Ethical permission was granted in Bangladesh and was not deemed necessary in Sri Lanka as the programme was conducted as part of routine clinical work. Written consent was obtained from parents to allow their experiences and feedback to be used for this research. Parents and clinicians also shared some reflections on their experiences and thoughts on the HAC programme.

Initial interviews and focus groups with parents suggest that the programme was well received by families in Sri Lanka and Bangladesh. Clinicians and group facilitators in Sri Lanka reflected that seven families participated despite a national fuel shortage and difficulties with transportation in 2022. Further, reflections from clinicians and group facilitators in Sri Lanka suggest that the structure was acceptable to parents as they found it helpful to describe their child's anxiety triggers in this way and valued the framework. All parents identified a SMART goal to work on, and by the end of the programme, parents were able to modify their SMART goal to include strategies such as ‘labelling my own emotions with my child to enable him to go to the grocery store three times a week during less crowded times’. This suggests that the structure of the groups was acceptable and the experiential exercises enabled parents to adopt strategies and amend their SMART goals. Further, in Bangladesh, parents reflected that they valued sharing their own methods and techniques with the group during the experiential exercises. Clinicians reflected that this supported the therapeutic process within the groups. Lastly, parents in Bangladesh reflected that being aware of their own emotion regulation strategies during the experiential exercises enabled them to consider how this affected their child's coping strategies.

Above all, preliminary findings suggest possible beneficial outcomes following feedback from parents who took part as well as the professionals who delivered the training. Parents and facilitators also reported the programme to be feasible and acceptable. The next steps include the completion of a larger trial of the programme. In addition to this we aim to create an adaptation of HAC to provide a psychoeducation programme for teachers in LMICs supporting autistic children experiencing anxiety.

## Data Availability

The data are not publicly available due to their containing information that could compromise the privacy of research participants.
